# Patient understanding and acceptability of an early lung cancer diagnosis trial: a qualitative study

**DOI:** 10.1186/s13063-018-2803-4

**Published:** 2018-08-04

**Authors:** Hayley C. Prout, Allan Barham, Emily Bongard, Rhiannon Tudor-Edwards, Gareth Griffiths, Willie Hamilton, Emily Harrop, Kerry Hood, Chris N. Hurt, Rosie Nelson, Catherine Porter, Kirsty Roberts, Trevor Rogers, Emma Thomas-Jones, Angela Tod, Seow Tien Yeo, Richard D. Neal, Annmarie Nelson

**Affiliations:** 1 Marie Curie Palliative Care Research Centre, School of Medicine, Cardiff University, Cardiff, UK; 2 Patient Representative, Cardiff, UK; 3 Centre for Health Economics and Medicines Evaluation, Bangor University, Bangor, UK; 4 Southampton Clinical Trials Unit, University of Southampton, Southampton, UK; 5 University of Exeter Medical School, Exeter, UK; 6 Centre for Trials Research, Cardiff University, Cardiff, UK; 7 School of Social and Community Medicine, University of Bristol, Bristol, UK; 8 Doncaster Royal Infirmary, Doncaster and Bassetlaw NHS Foundation Trust, Doncaster, UK; 9 School of Nursing and Midwifery, The University of Sheffield, Sheffield, UK; 10 Academic Unit of Primary Care, Leeds Institute of Health Sciences, Worsley Building (Room 10.35), Clarendon Way, Leeds, LS2 9NL UK

**Keywords:** Feasibility studies, Lung neoplasms, Patient preference, Primary healthcare, Qualitative research, Quality of life, Random allocation, Referral and consultation, Smoking, X-rays

## Abstract

**Background:**

The ELCID (Early Lung Cancer Investigation and Diagnosis) trial was a feasibility randomised controlled trial examining the effect on lung cancer diagnosis of lowering the threshold for referral for urgent chest x-ray for smokers and recent ex-smokers, aged over 60 years with new chest symptoms. The qualitative component aimed to explore the feasibility of individually randomising patients to an urgent chest x-ray or not and to investigate any barriers to patient recruitment and participation. We integrated this within the feasibility trial to inform the design of any future definitive trial, particularly in view of the lack of research exploring symptomatic patients’ experiences of participating in diagnostic trials for possible/suspected lung cancer. Although previous studies contributed valuable information concerning screening for lung cancer and patient participation in trials, this paper is the first to explore issues relating to this specific patient group.

**Methods:**

Qualitative interviews were conducted with 21 patients, comprising 9 who had been randomised to receive an immediate chest x-ray, 10 who were randomised to receive the standard treatment according to the National Institute for Health and Care Excellence guidelines, and 2 who chose not to participate in the trial. Interviews were analysed using a framework approach.

**Results:**

The findings of this analysis showed that altruism, personal benefit and the reassurance of not having lung cancer were important factors in patient participation. However, patients largely believed that being in the intervention arm was more beneficial, highlighting a lack of understanding of clinical equipoise. Disincentives to participation in the trial included the stigmatisation of patients who smoked (given the inclusion criteria). Although the majority of patients reported that they were happy with the trial design, there was evidence of poor understanding. Last, for several patients, placing trust in health professionals was preferred to understanding the trial processes.

**Conclusions:**

The integration of a qualitative study focusing on participant experience as a secondary outcome of a feasibility trial enabled exploration of patient response to participation and recruitment. The study demonstrated that although it is feasible to recruit patients to the ELCID trial, more work needs to be done to ensure an understanding of study principles and also of smoking stigmatisation.

**Trial registration:**

ClinicalTrials.gov, NCT01344005. Registered on 27 April 2011.

## Background

Lung cancer is the most common cause of cancer death in the United Kingdom, accounting for more than one in five cancer deaths. However, survival rates have not shown a great improvement in the last 40 years [1]. Furthermore, lung cancer survival rates in the United Kingdom and Ireland have been shown to be lower than the European average [2]. To address this inequity, several initiatives have been set up, such as the National Awareness and Early Diagnosis Initiative (NAEDI) [3] and the Together for Health Cancer Delivery Plan for the NHS for 2016 [4].

Options for earlier-stage diagnosis include the development of predictive biomarkers, getting general practitioners (GPs) to investigate symptoms more quickly (as in this trial), allowing GPs access to low-dose computed tomography [5], population screening programmes [6], and targeted public awareness campaigns to encourage earlier presentation of symptoms [7, 8].

### The ELCID trial

**Fig. 1:**
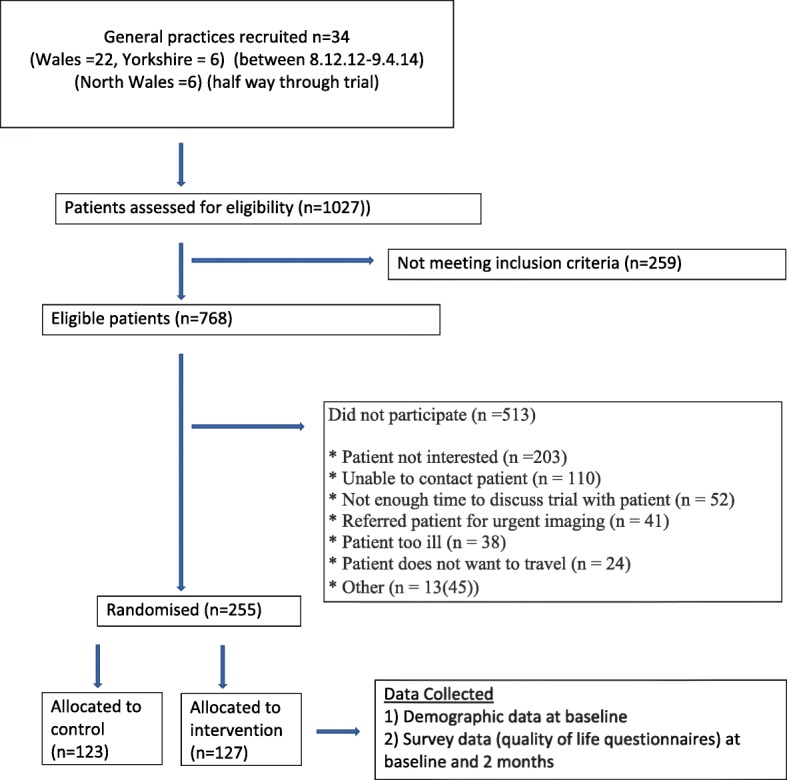
Recruitment and randomisation of patients

The ELCID (Early Lung Cancer Investigation and Diagnosis) feasibility clinical trial [9] is an NAEDI-funded trial examining the value of lowering the threshold for ordering a chest x-ray for suspected lung cancer symptoms in the primary care setting. Specific outcomes included evaluating trial design, materials and intervention and the training and recruitment of practices, including the recruitment and randomisation of patients (Fig. 1). The control group was investigated in accordance with contemporary National Institute for Health and Care Excellence (NICE) referral guidance (at the time), whereby they would undergo urgent referral with one of a number of chest symptoms present for more than 3 weeks. The trial intervention, which we have termed ‘Extra-Nice’, meant randomised patients would receive an urgent chest x-ray if they presented with one of a number of chest symptoms of any duration, smoked or were ex-smokers, and were over 60 years of age. Patients deemed eligible to participate in the trial included those over 60 years old who were either smokers or ex-smokers with a smoking history of 10 or more pack-years and who presented at a general practice with a new or altered cough of any duration or increased breathlessness or wheezing (whether or not associated with purulent sputum) [10].

This feasibility study was set up to inform the design of a large UK-wide clinical trial [9] of lowering the threshold for investigating patients presenting with symptoms of possible lung cancer. By lowering the threshold, it is hypothesised that clinical outcomes in lung cancer, as well as the cost-effectiveness of lung cancer diagnosis, may be improved. The study involved health economics, quality of life, and qualitative and quantitative methods in order to fully assess feasibility. This paper reports the qualitative findings.

### The ELCID qualitative study

Within the ELCID trial, an integrated qualitative study was carried out with the aim of exploring the feasibility of individually randomising patients to an urgent chest x-ray or not and to investigate any barriers to patient recruitment and participation. This integrated qualitative study is timely, considering the high mortality rates of lung cancer and the lack of research exploring symptomatic patients’ experiences of participating in trials for the possible diagnosis of lung cancer.

Our paper is one of only five which have explored patient experiences of lung cancer investigation and referral [11–13]. Banks and colleagues [12] investigated patient preferences for diagnostic testing for cancers including lung cancers using vignettes with primary care attendees. They found that participants expressed a preference for diagnostic testing at all risk levels and at levels below those stipulated by UK guidelines. Birt and colleagues [11] explored symptom appraisal and help-seeking decisions amongst patients with symptoms suggestive of lung cancer using in-depth qualitative interviews. They used this information to guide lung cancer awareness campaigns highlighting the importance of social networking and GP advice and monitoring. Banks and colleagues [13] highlighted minimal patient involvement in and understanding of referral decisions for investigation of lung and colorectal cancers. The qualitative interviews also brought to light GPs keeping dialogue non-specific and tending not to mention the possibility of cancer. Rankin and colleagues [14] used qualitative interviews and focus groups to explore the perspective of GPs and of their patients who had been placed on a lung cancer diagnostic pathway. Using the Model of Pathways to Treatment as a framework for analysis, they found that respondents felt that significant improvements should be made to health systems to improve experiences relating to diagnostic and pre-treatment intervals.

These studies highlight important information relating to symptom appraisal of lung cancer and diagnostic pathways to treatment. However, our study fills a need to explore patient experiences of specifically participating in a randomised controlled trial (RCT) relating to a potential lung cancer diagnosis. Because RCTs are considered of great importance in carrying out rigorous research [15–17], an insight into patient experiences of participating in an RCT to diagnose a possible lung cancer is of great importance.

### The benefits of embedded qualitative research

Recent integrated studies have generated valuable insight, such as patient preferences in a non-inferiority trial [18]; patient evaluations of trial principles, processes and practices in a non-placebo clinical trial for patients with advanced lung cancer [19]; issues of clinical equipoise and patient (mis)understandings in feasibility trials that experienced recruitment difficulties [20–24]; patient expectations of cancer diagnostics [12]; and participants’ understanding of complex trial processes in a stratified trial of personalised therapies [25]. Additionally, participant interview data highlighting trial processes in need of improvement may be used in real time to allow necessary protocol amendments in order to improve recruitment and retention of participants [26]. The ELCID qualitative study is reported in line with the guidelines set out in Consolidated Criteria for Reporting Qualitative Research (COREQ) [27].

### The aim of this paper

This paper reports the findings of the qualitative study that relate to participant understanding and acceptability of the trial and processes, such as recruitment and randomisation. By considering patient experience as a secondary outcome of the feasibility study, recommendations can then be made to inform the design of the ELCID phase 3 trial.

## Methods

### Study design

This was a multicentre, qualitative study which was embedded within a trial.

### Recruitment

The eligibility criteria for patients to be recruited into the main trial were the same for the qualitative interview study: patients over 60 years old who were either smokers or ex-smokers with 10 or more pack-years of smoking history and who presented at a general practice with a new or altered cough of any duration or increased breathlessness or wheezing (whether or not associated with purulent sputum). Therefore, all patients approached to take part in the ELCID trial (whether they chose to participate or not) were eligible to take part in the qualitative interview study, too. In addition to the eligibility criteria for the trial, however, patients were also required to be able and willing to discuss issues relating to their diagnosis, treatment, and quality of life and to be able to understand questions and speak English to the extent needed to participate in the interview. Patients who experienced any problems that affected their communication or comprehension were not included in the study. None of the patients, whether they participated in the trial or the interview study, were compensated for taking part. Patient characteristics are set out in Table 1.

**Table 1:** Participant characteristics of those interviewed for the ELCID Qualitative Study

(ID no.)	Group	Sex	Area recruited
1	Control	Female	South East Wales
2	Intervention	Female	South East Wales
3	Control	Female	South East Wales
4	Control	Female	South East Wales
5	Intervention	Female	South East Wales
6	Intervention	Female	South East Wales
7	Intervention	Male	South East Wales
8	Intervention	Male	South East Wales
9	Intervention	Female	South East Wales
10	Intervention	Male	South East Wales
11	Intervention	Female	South East Wales
12	Control	Female	South East Wales
13	Control	Male	South East Wales
14	Control	Male	South East Wales
15	Intervention	Female	South East Wales
16	Control	Male	South East Wales
17	Control	Female	South East Wales
18	Control	Female	South East Wales
19	Control	Male	South East Wales
20	Declined trial	Female	North Wales
21	Declined trial	Female	North Wales
22	Intervention (withdrawn)	Female	South East Wales

**Fig. 2:**
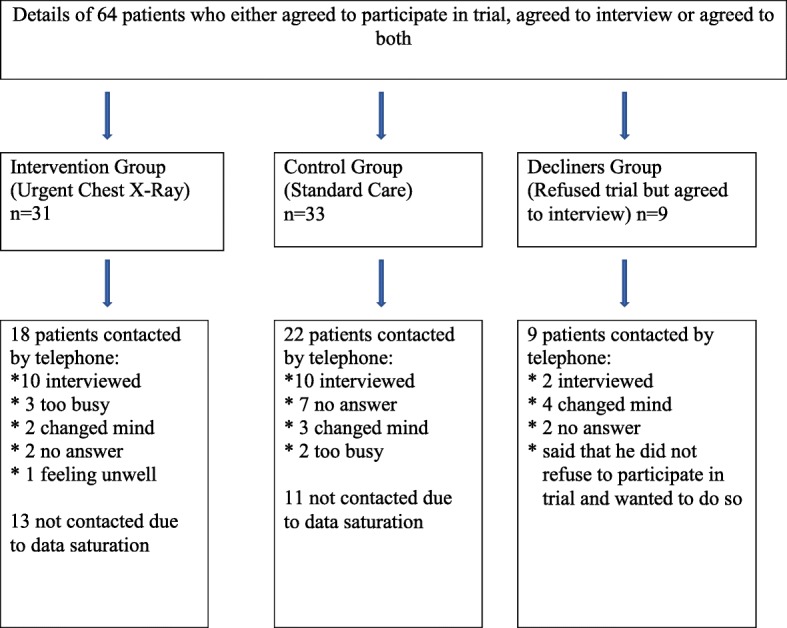
Recruitment of patients for the qualitative interview study

Patients were recruited into the study during their appointments with their GPs and at that time also indicated if they wanted to take part in the interview study, too (Fig. 2). Patients who declined consent for the trial were also asked if they would like to participate in the interview study, too. Sixty-four patients’ contact details were faxed to the qualitative researcher, and 22 of these patients were ultimately interviewed. The researcher telephoned the patients to determine an interview date. Patients were given at least 24 hours to decide whether to participate in the study. Consent was taken at the time and place of interview.

### Sampling and data collection

Thirty-four GP practice sites were open to recruitment. Purposive sampling was used according to the eligibility criteria set out above. Qualitative interviewing data were collected from 21 patients, 9 of whom had been randomised to receive an earlier chest x-ray, 10 of whom had been randomised to receive the standard treatment according to NICE guidelines, and 2 who chose not to participate in the trial. Recruitment for the interviews ceased when data saturation was reached [28]. However, the researchers managed to recruit only two patients for interview who declined trial participation. Although nine initially agreed to be interviewed, one patient changed their mind and seven were not contactable. Data saturation for this group was therefore not achieved.

### The qualitative interviews

The trial and the interviews were carried out simultaneously. The interviews, which lasted between 30 minutes and 1 hour, were carried out by a female researcher with a good knowledge of the healthcare system and experienced in qualitative interviewing. Although the researcher had a clinical background, she assumed the researcher role for the interviews. She did not hold any strong views about smoking and healthcare and remained neutral on issues that were discussed with the patient. She contacted the patients before visiting them in their homes and answered any questions that the patients asked.

Patients were given the choice of being interviewed either at their home or at their GP’s general practice. However, the patient ‘decliners’ were offered a telephone interview if they lived a long distance from the researcher’s place of work.

Three patients were accompanied in the interview by their spouse or partner, who was not additionally consented. For the most part, they stayed silent apart from their brief input, which was (1) to point out how nice a hospital was, (2) to agree with the patient that he did not need to wait long for an appointment, and (3) to clarify what the patient had said about the importance of the study. This input was not analysed and used for the paper.

The interview guide reflected the aim of the study: to explore the feasibility of individually randomising patients to an urgent chest x-ray or not and to investigate any barriers to patient recruitment and participation. Topics included the following:

- Feelings about being approached to participate in a trial concerning lung disease
- Reasons for taking part in the trial (or not)
- Experiences of taking part in the trial (if agreed to take part)
- Understanding of the trial design

The interview guide remained unchanged throughout data collection because no new topics were highlighted during the first few interviews.

#### Data analysis

Once all the interviews had been completed, they were uploaded via digital media for transcription using a standard operating procedure to ensure participant confidentiality. The anonymised transcripts were transcribed verbatim and uploaded to NVivo 10 software [29], and relevant extracts were isolated and coded. Data analysis was conducted via the Framework Analysis approach [30], which is suited to applied healthcare research situations where the aim of the study is to inform future practice, based on existing practice, rather than theoretical development. The analysis techniques include familiarisation (where the researcher becomes immersed in the data), developing a theoretical framework (where a hierarchical thematic framework is developed to classify and organise data into key themes, concepts, and categories), indexing (where the framework is applied to the original data transcripts and coded accordingly), charting (where each theme is charted using a table or matrix using summaries of the data), and mapping and interpretation (where the charts and data are examined for patterns and connections). Co-coding was carried out by the main qualitative researcher and a second qualitative researcher who worked in the same department. The second researcher carried out 10% of the interviews to ensure validity of the analysis and to verify interpretation. A thematic hierarchy was then produced. Any disagreements were resolved through discussion. Although data analysis commenced while the trial was ongoing, findings were too early to result in changes to the conduct of the trial.

## Results

These results reflect the aim of the study and the topics set out in the interview guide. They elucidate the reasons why patients decided to take part in the trial, their comprehension relating to trial design and processes, and their experiences of taking part in the trial and receiving a chest x-ray.

### Reasons for taking part in the trial

The majority of interview participants were happy to be approached for the study. Reported motivations for participating in the trial were mostly a combination of helping others, future generations, and themselves, with some patients stating that their family influenced their decision to participate.

#### Altruistic reasons for taking part

Several patients stated that they agreed to take part in the research in order to help their families. One patient said that it made her feel better to help her grandchildren and her husband;

*‘Well why I, I um done it [...] like I said is because of my grandchildren and my husband as well ’cos he had it, and I thought it was good thing for me to do like [...] and I felt I felt better because I done these things you know’. (Patient id2 female intervention arm)*

Respondents also made reference to participating for those whom they had lost. One respondent referenced her involvement as taking place in memory of a friend who had died of cancer:

*‘My friend had died from ovarian cancer right a few years before. […] I thought that [the trial] was something I was doing for her in a way if you know what I mean’.*

(Patient id3 female control arm)

Some patients also said that they explicitly wanted to help further medical research in the field of lung diseases:

*‘If it helps anything to do with you know medical research [...], yeah, I don’t mind helping, ay’.*

*(Patient id13 male control arm)*

Others expressed a moral duty to contribute in repayment for previous healthcare:

*‘It would be nice to give something back’.*

*(Patient id14 Male control arm)*

#### Personal gain

Patients made reference to taking part in the research in order to help themselves. One patient stated that although the main reason he participated was to help other people, he also thought that he would benefit from a chest x-ray by having a chest diagnosis:

*‘It’s a good cause try to help people I help myself like right […] they can find out what’s wrong with my chest, and I’m helping other people; that’s the reason why I’m doing it really’. (Patient id8 male intervention arm).*

Another patient stated that it was important to have tests in order to diagnose a problem early:

*‘To catch it early, you got a chance of living that much longer’.*

(Patient id15 female intervention arm)

### Concerns about taking part in the trial

A number of concerns about joining the trial were raised by participants and non-consenters. One patient pointed out that she did not want to take part in the trial but felt obliged to do so because the letter of invitation had originated from her GP (patient id15 female intervention arm). Her decision to participate was further based on the assurance that she would not be inconvenienced by extra travel. Another expressed anxiety about a poor medical outcome seemingly influenced by media reporting of a previous trial, and another patient was worried that she may have lung cancer:

*‘It [a large placebo trial] had quite a devastating effect on some of them [young people] [...]; that concerned me’.*

*(Patient id6 female intervention arm)*

*‘I was afraid I was very apprehensive about it because I had a chest infection and I thought, “Oh God, perhaps they think I’m a candidate for lung cancer or something”’.*

*(Patient id3 female control arm)*

One participant thought that she had been invited to take part in the trial because of her smoking status or history of smoking and the fact that she may have lung cancer, highlighting a smoking stigma:

*‘[I] felt a bit app, apprehensive first of all because I’m an ex-smoker [...], and I thought, “God, why have they picked on me?”’*

*(Patient id3 female control arm)*

The two participants who declined the trial gave different reasons for doing so, although both made connections to their own or others’ smoking behaviours. One trial decliner had previously been approached by health professionals multiple times about quitting smoking and assumed that she was again being approached:

*‘I thought it was about the stop smoking campaign, yeah, because, er, every time I’ve been to see anybody at the hospital or anything, everybody always says to me, “Would you like to stop smoking?”’*

*(Declined trial id20 Female)*

The other trial decliner stated that she was nervous anyway about being approached for the trial because her father, who had been a heavy smoker, had very recently died of lung cancer:

*‘I just lost me dad in November […], but he was a heavy smoker […], never went to the doctors’.*

(Declined trial id21 Female).

### Understanding and acceptance of trial design

A few patients showed some understanding of the trial. For example, one patient clarified that she understood the concept that not all patients would receive a chest x-ray:

*‘It’s random; only so many people can go [...], so it wouldn’t have bothered me at all [if I had not received an X-ray]’.*

*(Patient id5 female intervention arm)*

However, many patients were confused about the process of randomisation, with some believing that the process of being assigned to an arm of the trial was decided by the doctor in view of their past medical history or their smoking status:

*‘What I thought was information was fed into this computer that I smoked [...], and maybe that’s how they come to the decision that the people had it [the chest x-ray] or not’.*

*(Patient id6 female intervention arm)*

Another believed that he had not been assigned a chest x-ray, because the doctors had taken his medical history into account inasmuch as he had previously been for chest x-rays:

*‘I haven’t got to go for the x-ray, so obviously they must have seen something in my immediate past that doesn’t, um, merit me going for an x-ray, ’cos I’ve been for x-rays’.*

*(Patient id16 male control arm)*

### Understanding and acceptance of the control/standard care arm

It was apparent that several of the standard care patients had not adequately understood management allocation prior to agreeing to participate in the trial.

#### Misconceptions relating to the control/standard care arm

One standard care patient pointed out that he could not grasp an understanding of the purpose of the control arm:

*‘It’s just, I do not understand it [...] sounds pointless. Very pointless, actually.*

*(Patient id19 male control arm)*

Furthermore, many standard care patients believed that they were to have a chest x-ray well into the trial period. One patient stated that she had only entered onto the trial for the purpose of having a chest x-ray:

*‘Yes, because that was the whole idea in the beginning of deciding […] whether to go for it, I thought.[…] Other than that, to me, it [the trial] would be a bit pointless’.*

(Patient id12 female control arm)

Other standard care patients similarly described the benefits that they thought they would access by having the chest x-ray in terms of peace of mind and timely intervention if required:

*‘I just thought, oh, erm, lung disease research, it would be good. Peace of mind to know that my lungs were ok, [...] so I assumed that I would be x-rayed’. (Patient id17 female control arm)*

‘*It could have been caught it in time, then, and they possibly [could] do something about it’.*

*(Patient id13 male control arm)*

Another expected to have medical tests and could not understand why their health was not being investigated and all that they were doing was ‘*filling in forms’*:

*‘[I expected] tests of some sort. [...] But it’s just [...] filling in a couple of forms, and that’s it.*

*(Patient id19 male control arm).*

#### (non)-acceptance of control/standard care arm

Some patients felt that they would not have the best treatment if they were randomised to standard care, indicating a lack of understanding of trial equipoise. This was of particular concern for patients who believed that they needed a chest x-ray because of their symptoms. One patient even believed that being on the trial might mean a chest x-ray would not be taken even if clinically indicated during their routine care:

*‘I mean, I could be a really bad case, but because I wasn’t chosen’.*

*(Patient id17female control arm)*

Two patients on the intervention arm also indicated their hypothetical non-acceptance of the control arm, explaining that they would have possibly returned to see their GP to request an x-ray if they had been placed on the standard arm of the trial and were continuing to feel ill:

*‘Maybe I would have gone back and said, look, I need to have one if that’s how they do it’. (Patient id10 male intervention arm).*

However, although several of the standard care patients voiced an explicit preference for a chest x-ray and were disappointed not to receive one, many others accepted their allocation to the standard care or were indifferent to the issue:

*‘I’m indifferent on it, you know, either way I wouldn’t have minded [...] whether I had the x-ray or whether I don’t’.*

*(Patient id13 male control arm)*

Only one patient (standard care) stated a preference for the control arm, explaining that she would not have wanted a chest x-ray ‘because [of] fear’ of a potential cancer diagnosis (Patient id3 female control). She continued that she would prefer not to know “let’s just say ignorance is bliss” (Patient id3 female control). Even so, she would have accepted randomisation to receive a chest x-ray and continued on the trial.

### Patient experiences of the intervention arm (chest x-ray)

There did not appear to be any misunderstandings relating to the intervention or chest x-ray arm of the trial and those who had received their x-rays spoke positively about the process and outcome:

*‘Brilliant. That’s all I can say. [...] You know, it could have saved my life, well you know, lucky enough it didn’t have to, but it could have’.*

*(Patient id11 female intervention arm)*

Some patients also described how the results of the x-ray affected their smoking behaviour. One patient said that the opportunity of having a chest x-ray inspired him to improve his personal health, because he could quit smoking with the knowledge that he did not have cancer:

*‘It put my mind to rest to think, right, you’d better start getting yourself together now your chest is ok’.*

*(Patient id10 Male intervention arm)*

For another patient, however, it seems that a clear result of the chest x-ray would help to validate his smoking habit. He explained how he had smoked for 54 years and felt that smoking had not yet affected him, and a clear result would only confirm this:

*‘Everybody is telling me to give up smoking, but I’ve smoked about 54 years now. […] If this one comes up and it just confirms the first one [...], carry on smoking’.*

*(Patient id7 Male intervention arm)*

#### Receiving results

The patients who received a chest x-ray seemed to have no particular difficulties with the process of having the chest x-ray, stating that receiving the results of their chest x-ray was fast and efficient. One patient, however, received results that required further investigation, which understandably caused her significant anxiety:

*‘Terrible. It’s the waiting is the worst. I don’t think they get you in quick enough’.*

*(Patient id11 female intervention arm)*

#### Transport to hospital

Some patients stressed how easy it was to get to the hospital because certain surgeries provided taxi transportation, whereas others used public transport or otherwise with no issue:

*‘When they [the practice] said [hospital name 1], I said could I go to [hospital name 2], [...] and they said why is that? I said because parking up the [hospital name 1] is horrendous. Oh, don’t worry about that, she said, we’ll get you a taxi.*

*(Patient id11female intervention arm)*

*“I went up on my own; I wasn’t afraid.” (Patient id2 female intervention arm).*

However, a small minority of patients found the process of getting a chest x-ray difficult. One patient said that she had to pay for the parking costs and that using public transport would be too problematic (Patient id9 female intervention arm), whereas another patient stated that it would be too difficult to walk to the hospital in view of her ill health:

*‘The only thing is paying for the car to park. A nuisance. [...] The stupid little car park […], you couldn’t walk there. If you had to get a bus, it would be a major operation’.*

*(Patient id10 male intervention arm)*

### Patient evaluation of trial processes and documentation

#### Data collection processes

Some patients appeared to have a vague understanding of the course of the trial and what this entailed. However, several patients were unaware that they would receive a follow-up questionnaire after a few months, and one patient commented that she had not expected to be interviewed:

*‘I didn’t realise at the time that somebody was going to come, like yourself’.*

*(Patient id6 female intervention arm)*

#### Views on trial documentation and materials

In spite of the general lack of understanding of the trial processes, patients were generally positive about the trial documentation, with almost all of them saying that it was clear, informative and precise. Patients also seemed happy with the process of giving consent because they were not ‘pressured or pushed into it’ (Patient id9 female intervention arm) and had the option to withdraw from the study at any time:

*‘I hope I’ll see it through, but, um, as I say, if I’m allowed to say that’s it, I don’t want anymore, that’s good enough for me’.*

*(Patient id14 male control arm)*

In general, many patients preferred to receive information verbally. One stated that this is because she could ask questions if there was anything that she did not understand, whereas another thought she would remember it better if it was explained to her:

*‘I think it’s clearer if a person tells you because, um, when somebody’s talking to you, if you don’t understand something, you can ask them, [...] you know, whereas when you’re reading it [...], there’s no one to ask.’*

*(Patient id11 female intervention arm)*

Some preferred to receive information both verbally and in written format in order to compound knowledge, whereas others stated that they preferred receiving written information because it was more information and you could take it home to read. Some had no preference.

However, some patients reported problems with the documentation, namely data collection questionnaires. For example, one patient had difficulties regarding the clarity of a particular question asking whether she was anxious or depressed:

*‘There was one particular [question] [...]. It was, um, do you think you have or do you think you’re anxious or depressed? [...] I said to the lady, you know, how do I answer this, because I am anxious but I’m not depressed?’*

*(Patient id11 female intervention arm)*

Two patients pointed out that they thought that the patient questionnaire was intrusive:

*‘I thought maybe one or two could have been a bit intrusive [...], but not to me, you know, I don’t mind […]. Other people may think you know some of the questionnaire was a bit intrusive like [...], but, er, as far as I was concerned, it was, you know, straightforward’.*

*(Patient id13 male control arm)*

#### Preference for limited information and trusting in health professionals

Several patients preferred to take a more passive role in the trial, explaining how they did not feel the need to understand the inner workings of the trial because they felt able to trust their doctors and other practice staff to do right by them:

‘*I felt that you would understand everything, whoever did the trials [...], and really there was no need for me to [...]really understand fully’. (Patient id12 female control arm)*

Likewise, another patient said that he would accept whichever arm of the trial to which he was randomised, owing to his trust in the research team:

*‘You people [...] are running the show, you know, captain of the ship, so that’s it, you obey’.*

*(Patient id7 male intervention arm)*

## Discussion

We carried out 21 qualitative interviews as an integral component of the ELCID feasibility clinical trial in order to explore patient response to participation and recruitment to a trial associated with lung cancer diagnosis and a chest x-ray referral. The results showed that altruism, personal benefit and the reassurance of not having lung cancer were important factors in patient participation. However, patients largely believed that being in the intervention arm was more beneficial, highlighting a lack of understanding of clinical equipoise. Disincentives to participation in the trial included the stigmatisation of patients who smoked, owing to the inclusion criteria. Although the majority of patients reported that they were happy with the trial design, there was evidence of poor understanding. Last, for several patients, placing trust in health professionals was preferred to understanding the trial processes.

### Reasons for taking part in the trial

Our study highlighted that altruism and perceived medical benefit, including reassurance of not having lung cancer, were motivations to take part in this trial. Others have similarly identified medical benefit to be a primary motivation for taking part in trials, with participants joining trials in the hope of accessing treatments which might help them [18, 19, 31]. In this study, this attitude related to a belief amongst many participants that an early chest x-ray is important for earlier diagnosis of cancer. This may mirror a more widespread public attitude towards tests, as suggested in the study by Banks and colleagues [12] noted above, which found a clear preference for testing in spite of relatively low risk levels.

What this also demonstrates, however, is a lack of understanding or acceptance of trial equipoise amongst many participants and possible diagnostic misconception, with some patients apparently joining the study because of a perceived clinical need and assumed benefit from receiving the intervention. Previous research has similarly highlighted patient difficulties in understanding or accepting clinical equipoise, for reasons such as patient/public beliefs about particular treatment options (as indicated above) [22, 23, 32], perceived clinical preferences for particular treatment arms [19, 21, 23, 24, 33], and an orientation towards trusting the experts and their expertise when making treatment decisions (as opposed to accepting clinical uncertainty and the related need for an RCT) [19–21, 23, 24, 34]. Indeed, Mills and colleagues [21] pointed out that clinical equipoise or the consensus by experts that there are no merits to either treatment being tested had an effect on participation in their trial of treatments for prostate cancer. They found that those patients who did not accept the ‘equipoise’ in the trial were less likely to participate.

### Concerns over taking part in the trial

Many participant misunderstandings of the nature of the ELCID trial were closely related to attitudes and beliefs regarding the study. Two patients who had agreed to be interviewed for the ELCID interview study chose not to participate in the trial because of a perceived smoking stigma. It is therefore possible that there were other potential participants who chose not to take part in the trial because of the associated smoking stigma and a belief that the trial involved a smoking cessation intervention. This reinforces findings of the das Nair and colleagues study [35], which highlighted stigmatisation related to smoking as a barrier to possible recruitment to a lung cancer screening trial.

A small number of patients reported cancer anxiety upon receiving the initial letter of invitation to participate in ELCID. Banks and colleagues [13] also noted that anxiety was raised only when patients had been referred for a chest x-ray and there was a lack of information given to the patient.

### Understanding and acceptance of trial design

There have been no studies exploring participant understanding of diagnostic lung cancer trials. Although there were some patients who expressed an understanding of randomisation and the trial processes, the majority did not, with a number of patients perceiving that their allocation was based on clinical assessment, as also noted in other studies [23]. Our study shows that many patients’ expectations are misinformed or confused. This was despite significant patient and public involvement input into the design of materials and ethics committee processes. The general literature concerning patient understanding of RCTs provides further evidence of participants’ poor understanding of trials and key principles such as randomisation and equipoise [22, 23, 31, 36–38]. In a recent systematic review and meta-analysis of the quality of informed consent of clinical trials, Tam and colleagues [38] found some aspects of the trial were not understood by participants. Whilst there was understanding of the benefits of the voluntary nature of the study, the concepts of randomisation and placebo were not understood. They point out that this lack of understanding had not changed over 30 years.

### Patient evaluation of trial processes and documentation

Our study revealed the extensive influence of healthcare professionals in information-giving and decision-making. This could partly be the reason for participants’ not understanding the information, because they may be relying on health professionals rather than themselves to take more notice of the content of the trial. Banks and colleagues [13] also noted that patients who had been referred for a chest x-ray expected the GP rather than themselves to make this decision to have this test.

Locock and Smith [31] carried out qualitative interviews with participants who had previously taken part in clinical trials for a range of different conditions. They, too, found that trust in the medical and nursing staff was a common theme, with some participants stating that they had not really read the information sheet but preferred to put their trust in discussing the trial with the clinical team instead. Alternatively, Doyal [39] pointed out that some patients have problems with understanding clinical information and therefore do not wish to participate in making decisions.

### Strengths and limitations of the study

Our study is the first to explore the experiences of symptomatic patients who are participating in trials for possible lung cancer. Although previous studies contributed valuable information concerning screening for lung cancer and patient participation in trials, this paper explored issues relating to this specific patient group. It is one of only four which have explored patient experiences of lung cancer investigation and referral [11–13]. A further strength of this study is that not only patients from the intervention and control arms of the trial but also those who refused to take part in the trial were interviewed.

A limitation of the study is that the two patients who declined to take part in the main trial highlighted important information regarding smoking stigma. A larger sample within this decliners group could have generated more information regarding why patients refused to participate in the ELCID clinical trial. A further limitation could be gender and geographical biases. There were double the amount of females who participated in the interview study (females, *n* = 14 [1 withdrawn]; males, *n* = 7). Also, all patients who participated in the trial and the interview study were from southeastern Wales, and none were from North Wales and Yorkshire. The two patients who declined the trial were from North Wales.

## Conclusions

The integration of a qualitative component focused on participant experience as a secondary outcome of a feasibility trial has enabled exploration of patient response to participation and recruitment. This has demonstrated that although it is feasible to recruit patients to the ELCID trial, more work needs to be done to ensure an understanding of study principles and also of smoking stigmatisation. Recommendations for the next phase of the ELCID diagnostic trial include the following:

- To motivate patients to take part in future lung cancer diagnostic trials, importance needs to be placed on highlighting the possibility of helping others and advancing medical science.
- Patients should be supported to take the necessary time to ensure understanding of patient information sheets before signing consent, especially with regard to clinical equipoise and that they will not necessarily benefit from participation.
- Patients should be assured that the aim of the study is not to stop smoking, because it seems that this may limit recruitment owing to smoking stigmatisation.
- Consideration should be given to a shared decision-making approach for those patients who are less motivated to make decisions on their own behalf.
- Patients should be reassured that participation in the trial should cause the patient the least amount of inconvenience, especially in terms of travel necessities.

These recommendations should be considered for future trials concerning lung diagnostics. They can also be used when considering any trial and the need to support patients to understand the study in which they are consenting to participate.

## Abbreviations

ELCID: Early Lung Cancer Investigation and Diagnosis
GP: General practitioner
NAEDI: National Awareness and Early Diagnosis Initiative
NICE: National Institute for Health and Care Excellence
RCT: Randomised controlled trial
